# Effect of West Indian Bay Leaf (*Pimenta racemosa*) and Turmeric (*Curcuma longa*) Essential Oils on Preserving Raw Chicken Breasts

**DOI:** 10.17113/ftb.62.02.24.8155

**Published:** 2024-06

**Authors:** Che John, Rohanie Maharaj

**Affiliations:** Department of Chemical Engineering, The University of the West Indies, St. Augustine Circular Road, St. Augustine, Trinidad and Tobago

**Keywords:** essential oil, West Indian bay leaf, turmeric, novel preservatives, chicken breast

## Abstract

**Research background:**

While the use of chemical preservatives in meat may appear to be tremendously advantageous, they have long been purported to increase the risk of incidence of certain types of cancers. Consequently, many people have opted for minimally processed alternatives. This consumer shift has placed substantial pressure on the food industry to implement more natural alternatives to these synthetic preservatives in the meat industry. Research on plant extracts as potential agents for food additives is increasing. The bioactive components present in West Indian bay leaf and turmeric essential oils have a promising potential for use as novel, green preservatives in the meat industry.

**Experimental approach:**

Raw chicken breast samples (28 g) were each treated with different volumes (0.5, 1 and 1.5 mL) of the essential oil of West Indian bay leaf or turmeric or their mixture (1:1 to make up a final volume of 0.5, 1 and 1.5 mL). Physicochemical, microbiological and sensory evaluations were performed on the fresh and treated samples stored for 14 days at 4 °C.

**Results and conclusions:**

The West Indian bay leaf oil had a higher extraction yield and total phenolic content, while the turmeric oil had a higher total flavonoid content. The most effective treatments, compared to the control, significantly (p<0.05) minimized the pH increase by 13.9 % (1.5 mL bay leaf oil), reduced texture loss by 44.8 % (1.5 mL oil mixture) and reduced protein loss by 98.9 % (1 mL bay leaf oil). Most treated samples had reduced microbial loads, with the turmeric oil showing the highest efficacy against lactic acid bacteria, yeasts and moulds. Treated samples had significantly higher (p<0.05) sensory scores than the control on the final day of storage, with the 1.5 mL oil mixture proving to be the most effective, as the storage life of the chicken breast sample was extended by 6 days.

**Novelty and scientific contribution:**

This study has shown for the first time that the essential oil from turmeric and West Indian bay leaf can extend the shelf life of raw chicken breast and highlights the potential of the oil as natural preservative agents in lieu of synthetic alternatives.

## INTRODUCTION

Raw meat with its high water content, nutrients and almost neutral pH is an ideal environment for microbial proliferation (*1*). Preservatives in meat such as nitrites and nitrates inhibit microbial growth through their innate bactericidal effect (*2*). Fresh chickens are injected with sodium acetate or sodium lactate at slaughter to prolong shelf-life through their antimicrobial effect (*3*). Preservatives such as nitrates appear to be beneficial, but their unregulated use and overconsumption can be detrimental to consumer health. Nitrates used in meat processing can react with available secondary amines after reduction to nitrites and produce carcinogenic nitroso compounds (NOCs) (*4*, *5*). As these harmful effects have become widely known, consumers have begun to limit their intake of highly processed meat and instead focused on minimally processed alternatives (*6*). This shift in consumer awareness is forcing the food industry to respond and use more natural preservatives.

Some plant essential oils have been shown to have quite potent antimicrobial properties, such as lemon (*Citrus limon*) and eucalyptus (*Eucalyptus globulus*), which are used in food coatings and active food packaging, as they protect against both spoilage and pathogenic microbes (*7*). While the antibacterial and preservative properties of the more popular aromatic plants and herbs have led to their increasing use in the food industry worldwide, this is not the case with the use of traditional local herbs and spices in the small island developing states of the Caribbean.

*Pimenta racemosa*, commonly known as West Indian bay leaf in Trinidad and Tobago, is a tall, aromatic, arboreal plant native to both the Caribbean and northwestern South America (*8*). The volatile compounds in the essential oil of *Pimenta racemosa* leaves consist of various phenols, monoterpenes, sesquiterpenes, diterpenes and esters (*9*). Eugenol has also been shown to be the most abundant of these volatile compounds, ranging from 60.4 to 82.9 %, and is thus the source of most of the antibacterial potential (*8*). Eugenol, which is extracted from the essential oil of cloves and cinnamon leaves, has strong antibacterial and insecticidal properties (*10*).

Turmeric (*Curcuma longa*) is a perennial plant with underground rhizomes that are predominantly oblong, ovate and short-branched (*11*). It belongs to the Zingiberaceae family, and is a close relative of the better known ginger, which has similar physical properties and strong antioxidant, antibacterial and anti-inflammatory properties (*12*). The numerous bioactive compounds found in turmeric, such as sesquiterpenes, ketones, tumerone, zingiberene, cineole and various curcuminoids, are responsible for these biological properties. Previous research has also shown that curcumin is the main curcuminoid and the primary phytochemical responsible for the biological functions of turmeric (*11*).

While the antimicrobial effect of these two essential oils has already been demonstrated, the effect of West Indian bay leaf essential oil as a natural preservative and its effect in combination with turmeric essential oil has not yet been investigated. In this study, some quality indicators of the essential oil extracted from West Indian bay leaf and turmeric as natural preservatives in raw chicken breast samples were investigated.

## MATERIALS AND METHODS

### Collection and preparation of plant material

Fresh West Indian bay leaves were collected from a single large tree on the St. Augustine campus of the University of The West Indies, Trinidad and Tobago. Approximately 3 kg of the leaves were carefully harvested and rinsed thoroughly under cold running tap water to remove all debris and organisms. The leaves were then placed on a kraft drying paper (150 cm×50 cm) to air dry for 10 days. After that, 0.95 kg of the leaves were used for the essential oil extraction. Fresh turmeric rhizomes (9 kg) were obtained from a Farmers’ Market in Macoya, Trinidad and Tobago. The rhizomes were thoroughly rinsed under cold running tap water to remove soil and debris and then air-dried for 5 days until they reached approx. 82.2 % of their initial mass. After drying, the rhizomes were cut into 0.2 cm thin slices using a Hobart slicer (model 1612; Hobart Corporation, Troy, OH, USA) and then partially crushed with a wooden mallet to increase the surface area and facilitate the extraction of the essential oil by steam distillation.

**Fig. S1:**
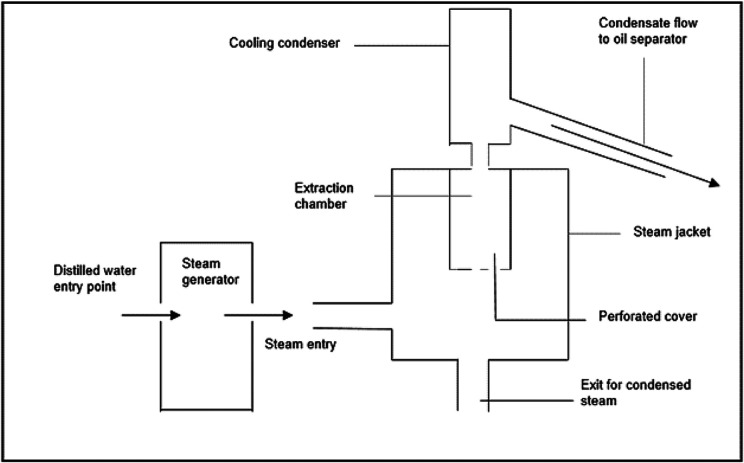
Diagram of the steam distillation apparatus

The essential oil was extracted from the West Indian bay leaf and turmeric by steam distillation in a pilot plant (Fig. S1). The dried leaves (0.95 kg) were placed in a steam distillation drum and the distillation process was left to run for three hours. The same procedure was used for the sliced and partially crushed rhizomes (7.4 kg). After the run time of both extractions had elapsed, the essential oil obtained was collected in 40-mL amber bottles and immediately refrigerated at 4 °C until needed for the experiments.

### Total phenolic content and total flavonoid content

The Folin-Ciocalteu colourimetric method (*13*) was used to measure the total phenolic content (TPC) of the extracted essential oil. The absorbance was measured at 765 nm and the TPC of the extracted essential oil was expressed in mg of gallic acid equivalents (GAE) per mL of sample.

The aluminium chloride colourimetric test (*14*) was used to determine the total flavonoid content (TFC) of each essential oil. The absorbance was measured at 415 nm and expressed in mg of quercetin equivalents (QE) per mL of sample. A UV-Visible spectrophotometer (Thermo Scientific Evolution 60S; Thermo Fisher Scientific, Waltham, MA, USA) was used for both TPC and TFC determinations. The chemicals used for the analyses were of reagent grade and purchased from Sigma-Aldrich, Merck (St. Louis, MO, USA).

### Preparation and treatment of samples

Raw bone-in chicken breasts (2.0 kg) were purchased from a poultry depot in St. Augustine, Trinidad and Tobago, and were immediately placed in an insulated cooler before arrival at the laboratory. After rinsing with potable water, the breasts were cut to obtain two main sets of samples, one triplicate set of eleven 28-gram samples each (for quantitative analyses), and another set of ten 28-gram samples (for sensory analysis). The ten samples from the set for sensory analysis were labelled as follows: turmeric 0.5 mL, turmeric 1 mL, turmeric 1.5 mL, West Indian bay leaf 0.5 mL, West Indian bay leaf 1 mL, West Indian bay leaf 1.5 mL, mixture 0.5 mL (West Indian bay leaf 0.25 mL and turmeric 0.25 mL), mixture 1 mL (West Indian bay leaf 0.5 mL and turmeric 0.5 mL), mixture 1.5 mL (West Indian bay leaf 0.75 mL and turmeric 0.75 mL) and stored control (untreated sample). Ten samples from the quantitative sample set were labelled similarly, with the last, eleventh sample serving as a fresh control breast sample.

Each sample was placed in an individual aluminium foil sheet (12 cm×12 cm) and the amount of the corresponding essential oil was aseptically applied to the entire surface of the samples using a micropipette to ensure uniform application as indicated on the sample label. Each sample, including the control, was then wrapped in the aluminium foil and placed in a labelled container and stored at 4 °C for a period of 14 days. The remaining chicken breasts, which were neither treated with the essential oil nor refrigerated, served as fresh, unstored control samples (day 0 control).

### Physicochemical assessment of samples

#### Colour

The colour values of the fresh samples (day 0 control) were recorded with a Konica Minolta Chroma Meter (CR-400; Tokyo, Japan) in the CIELAB colour space values of L*, a*, b* and similarly this process was repeated on day 14 for the treated and stored control samples at 4 °C.

#### Texture

Texture was expressed as hardness (N) of the chicken breast samples using the QTS 7113 texture analyser (CNS Farnell, Leeds, UK). The sample was positioned on the platform of the texture analyser and a full profile was analysed using a TA9 needle probe (1.5 mm diameter) at a constant speed of 1.0 mm/s until a predetermined distance of 15 mm was reached.

#### pH

A mass of 5 g sample was placed in a sterile stomacher bag and 50 mL of distilled water were added. The stomacher bag was then placed in a stomacher blender and the sample was allowed to homogenise for 1 min. The bag contents were transferred to a clean 100-mL beaker and the pH of the homogenate was measured using the pH 211 microprocessor pH meter (Hanna Instruments, Woonsocket, RI, USA).

#### Moisture content

The moisture content of the fresh and refrigerated chicken breast samples (2 g of cut samples) was determined using the convection oven method (Thelco 130D; Precision Scientific, Denver, CO, USA). Samples were placed in a preheated oven at 198 °C for 1.20 h, after which they were cooled to ambient temperature in a desiccator and then weighed. The mass fraction of moisture in each sample was determined using the following equation:


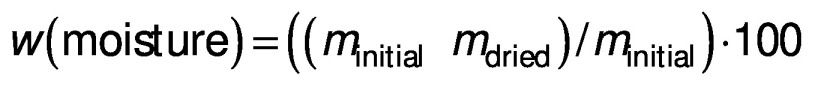
 /1/

where *m*_initial_ is the mass before drying (in g) and *m*_dried_ is the mass after drying (in g).

#### Protein content

The protein content of the samples was determined using the Kjeldhal method (*15*). A Gerhardt digestion and distillation system (Gerhardt Analytical Systems, Königswinter, Germany) was used, and the nitrogen and subsequent protein percentages were calculated using the following equations:


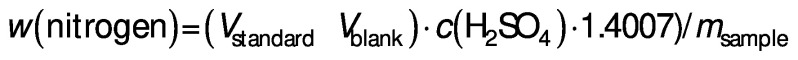
 /2/

where *V*_standard_ and *V*_blank_ are in mL, *c*(H_2_SO_4_) in mol/L and *m*_sample_ in g, and:


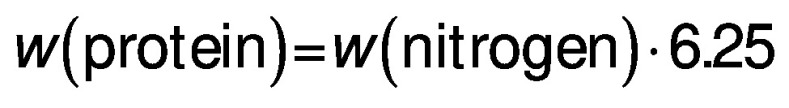
 /3/

where 6.25 is specific factor for the conversion of nitrogen to protein content in meat.

### Microbiological evaluation

The microbiological analyses of the samples were conducted according to the standard enumeration procedures for total plate count, total yeast and mould and lactic acid bacteria as outlined in the Bacteriological Analytical Manual (BAM) (*16*).

#### Preparation of culture media

Dichloran Rose-Bengal chloramphenicol (DRBC); De Man, Rogosa and Sharpe (MRS) and total plate count agars (Oxoid Limited, Hampshire, UK) were used to enumerate the yeasts and moulds, lactic acid bacteria, and the total number of aerobes, respectively, in each sample (fresh and refrigerated). A volume of 20 mL aliquot of each agar, prepared according to the manufacturer’s instructions, was poured into 25 mL VWR 100 mm×15 mm Petri dishes and allowed to set at 4 °C for 24 h before refrigeration to subsequently plate the 11 samples.

#### Plating of samples and colony enumeration

A mass of 5 g of each cut meat sample (treated and control) was homogenised with 45 mL of diluent (0.85 % NaCl) and used to prepare four serial dilutions (10^-1^, 10^-2^, 10^-3^ and 10^-4^). Using the spread plate method, 0.1 mL of each of the four prepared sample dilutions was added to a separate agar plate. The MRS and DRBC plates were incubated at 35 and 25 °C for 72 h, respectively, while the total plate was incubated at 35 °C for 48 h. The plates were then counted using a Quebec Darkfield colony counter (Reichert, Inc., Depew, NY, USA). Plates with more than 300 colonies were considered too numerous to count (TNTC), while those with less than 30 colonies were considered too few to count (TFTC). The average number of colonies was used to calculate the colony forming units per gram of the initial 5-gram sample.

### Sensory evaluation

The treated and control chicken breast samples were evaluated using a 7-point hedonic scale that ranged from extremely unacceptable (1) to extremely acceptable (7) for the attributes of odour, colour and appearance at the end of the 14-day storage, while overall acceptability was analysed every 48 h over the 14-day storage period. The overall acceptability parameter was used to indicate storage life quality with average sensory scores of less than 3.0 interpreted as spoilage of the sample. The treated samples and control were evaluated at 10 am by a panel of 30 semi-trained individuals (15 males and 15 females aged 18-22) made up of students from the University of the West Indies, who were not permitted to touch the samples, but only to visually observe and smell them. The samples were each assigned a random three-character code, wrapped in aluminium foil and placed in uniform, odourless plastic containers at room temperature ((25±1) °C).

### Statistical analysis

Statistical analysis of the data was carried out using SPSS statistical software v. 29.0 (*17*) to conduct a one-way ANOVA and Dunnett’s multiple comparison tests to determine whether treatment differences were significant. The Tukey’s multiple range test was also used to compare the results of each treatment group to determine if the differences between treatments were significant. Significant differences were found at p<0.05.

## RESULTS AND DISCUSSION

### Yield and phytochemical content of essential oils

The steam distillation technique used in this study resulted in the extraction of 20 mL of West Indian bay leaf and 15 mL of turmeric essential oil, corresponding to a yield of 0.02 and 0.002 %, respectively. The total phenolic content (TPC), expressed as gallic acid equivalents (GAE), and total flavonoid content (TFC), expressed as quercetin equivalents (QE), of turmeric essential oil were determined to be (2.6±0.4) and (3.14±0.03) mg/mL, respectively, while the TPC and TFC of bay leaf essential oil were (7.3±0.2) and (2.2±0.1) mg/mL, respectively. Phenols and flavonoids are the main classes of the phytochemical components and their total content is a good indicator of how effective an essential oil is as an antimicrobial agent (*18*). While not many studies have been conducted on the total phenolic content of West Indian bay leaf essential oils, the phenolic content, expressed as GAE, of turmeric essential oil was at the lower end of the range of 2.80−13.40 mg/mL found in previous studies (*19*). With the values obtained for both the total phenolic and flavonoid contents of the West Indian bay leaf and turmeric essential oils, it was expected that the bay leaf oil would have a higher antimicrobial activity and thus be a better preservative as the total phytochemical content (TPC and TFC) was higher than that of the turmeric oil.

### Effect of essential oil on sample pH

During meat spoilage, the breakdown of proteins and the formation of amines and ammonia from amino acids cause the characteristic increase in the pH of meat, which often reaches values of up to 8.5 (*20*). The results summarised in Table 1 show that all treated samples had significantly lower (p<0.05) pH values on day 14 than the refrigerated control. These findings are consistent with the results of previous studies that have shown that the presence of essential oils can slow the increase of pH of meat during spoilage (*21*). Treatment with 1.5 mL West Indian bay leaf essential oil was the most effective, as the treated sample had the lowest pH, 13.9 % lower than the refrigerated control. This observation could be a direct result of the differences in the TPC and TFC values of turmeric and West Indian bay leaf essential oil. Kaur and Mondal (*22*) have shown that plant species with a higher phenolic content had a stronger antibacterial effect than others, even if their TFC was similar. This supports the results of this study, as the higher TPC of the bay leaf essential oil enabled it to better suppress the growth of pH-altering proteolytic bacteria than the turmeric essential oil. This resulted in significantly lower (p<0.05) pH values of the West Indian bay leaf essential oil samples of 1 and 1.5 mL than of the respective turmeric essential oil samples. No significant difference (p>0.05) was observed between the treatments when 0.5 mL of the oil was used. Furthermore, when the volume of bay leaf essential oil was increased from 1 to 1.5 mL, the pH decreased significantly (p<0.05) as the samples were likely exposed to higher amounts of phenols, which further inhibited the growth of proteolytic bacteria.

**Table 1:** Physicochemical parameters of fresh control sample (day 0 at 28 °C), refrigerated control (day 14) and treated (day 14) chicken breast samples stored at 4 °C

Sample treatment	pH	Hardness/N	*w*(protein)/%	*w*(moisture)/%	L*	a*	b*
*V*/mL Turmeric oil							
0.5	(6.78±0.01)^d^	(0.58±0.08)^cd^	(20.9±1.2)^ab^	(23.8±0.3)^ab^	(67.88±0.02)^b^	(12.54±0.02)^d^	(17.15±0.01)^cde^
1	(6.90±0.01)^e^	(0.79±0.07)^ef^	(21.8±0.8)^ab^	(24.1±1.5)^ab^	(72.2±0.1)^d^	(12.09±0.03)^c^	(16.72±0.03)^bcd^
1.5	(7.01±0.03)^f^	(0.80±0.06)^ef^	(22.3±0.6)^abc^	(25.0±1.0)^ab^	(70.8±0.6)^c^	(15.23±0.07)^h^	(19.7±0.4)^g^
West Indian bay leaf oil							
0.5	(6.82±0.01)^de^	(0.43±0.02)^b^	(21.3±0.3)^ab^	(24.4±1.0)^ab^	(67.82±0.02)^b^	(14.02±0.01)^f^	(18.28±0.01)^f^
1	(6.62±0.01)^c^	(0.72±0.08)^de^	(2498±0.9)^c^	(25.6±0.5)^ab^	(75.65±0.06)^e^	(11.93±0.01)^b^	(16.36±0.03)^bc^
1.5	(6.32±0.02)^b^	(0.76±0.02)^ef^	(22.50±0.05)^bc^	(26.3±0.2)^ab^	(85.48±0.05)^g^	(12.60±0.05)^de^	(18.6±1.1)^fg^
Oil mixture							
0.5	(6.74±0.01)^d^	(0.51±0.02)^bc^	(20.9±1.2)^ab^	(23.3±0.6)^a^	(67.81±0.04)^b^	(12.68±0.01)^e^	(15.64±0.01)^b^
1	(6.40±0.01)^b^	(0.69±0.04)^de^	(22.6±0.8)^bc^	(26.4±0.2)^ab^	(85.3±0.3)^g^	(14.80±0.05)^g^	(18.16±0.01)^ef^
1.5	(6.36±0.01)^b^	(0.88±0.02)^af^	(22.1±0.8)^abc^	(28.5±1.6)^b^	(83.9±0.2)^f^	(11.00±0.02)^a^	(16.33±0.02)^bc^
*t*/day							
0 (fresh control)	(5.91±0.02)^a^	(0.95±0.03)^a^	(24.94±0.08)^c^	(78.3±2.7)^c^	(72.22±0.09)^d^	(19.62±0.04)^i^	(14.4±0.2)^a^
14 (refrigerated control)	(7.34±0.08)^g^	(0.49±0.02)^bc^	(19.3±0.2)^a^	(23.3±0.4)^a^	(60.24±0.08)^a^	(10.9±0.1)^a^	(17.8±0.3)^def^

Different letters in superscript in the same column indicate significantly different values (p<0.05)

This trend was not observed in the turmeric samples, as the pH conversely increased significantly (p<0.05) with the increase of the treatment volume, indicating that higher proliferation of proteolytic bacteria occurred with increasing volume, albeit to a lesser extent than in the refrigerated control.

The samples treated with the mixture of essential oils had significantly lower (p<0.05) pH values than the refrigerated control, and both the 1 and 1.5 mL had a similar effect to the 1.5 mL bay leaf sample. Individually, West Indian bay leaf essential oil was more effective than turmeric essential oil, but when used in mixture, a synergistic effect was observed. The sample treated with a mixture of 1 mL (0.5 mL of each bay leaf and turmeric essential oil) had a significantly lower pH (p<0.05) than the samples treated with the two individual volumes of 0.5 mL, while the 1.5 mL mixture was as effective as 1.5 mL of bay leaf essential oil (highest volume treatment). The results show that less of each oil was required in the mixture to achieve a similar or even better effect than when the oils were used individually.

When mixed essential oils are used, their effects can sometimes be synergistic and lead to an enhanced antibacterial response (*23*). The enhanced effect of the mixtures of 1 and 1.5 mL of essential oils could lead to a stronger inhibition of the proteolytic bacteria that typically alter the pH in meat. No significant difference (p>0.05) was observed when the volume of mixed oils increased from 1 to 1.5 mL, suggesting that the synergistic effect probably decreased as the maximum efficacy peaked at the 1 mL. It is possible that the increased volume of the less effective turmeric oil had a diminishing effect on the efficacy of bay leaf oil.

### Effect of essential oil on sample texture

As expected, the refrigerated untreated sample showed the highest decrease in average hardness, which was almost 50 % compared to the fresh control (Table 1). During spoilage, autolytic breakdown of protein myofibrils and the effects of biofilm formation contribute to the softening of the meat texture (*24*). In particular, the proliferation of bacterial species such as *Lactobacillus* spp., *Leuconostoc* spp. and *Pseudomonas* spp. probably caused the formation of biofilm on the meat surface, which had a negative effect on the texture (*25*).

All treatments with the essential oil above 0.5 mL significantly reduced (p<0.05) softening compared to the refrigerated control, indicating that the 0.5 mL does not have enough antibacterial potency to delay the textural changes in the samples. Only at a volume of 1 mL of each treated sample was such an effect observed, where more bioactive phytochemical elements were present to slow down the changes in texture. Furthermore, while an increase from 0.5 to 1 mL resulted in a significant difference (p<0.05) in the hardness of the samples, no further significant effect (p>0.05) was observed when the volumes were increased from 1 to 1.5 mL, except in the case of mixed oil treatment, where the 1.5 mL sample retained significantly greater (p<0.05) hardness than the 1 mL sample. Additionally, apart from the previously determined ineffective volume of 0.5 mL, no statistically significant difference (p>0.05) was observed between the turmeric and bay leaf oil treatments.

The hardness of the samples treated with 1 and 1.5 mL was significantly higher (p<0.05) than of the stored control, and significantly lower (p<0.05) than the hardness of the fresh sample, except for the sample treated with 1.5 mL of essential oil mixture, which retained 92.7 % of the total hardness of the initial fresh sample. Thus, while no synergistic effect of the single oils was observed at the other lower volumes, there was a synergistic effect at the highest volumes used.

### Effect of essential oil on protein content

The protein mass fraction of the fresh sample was close to the previously reported values of 22.8−23.3 % (*26*). Due to microbial action, protein oxidation and autolytic processes occur, whereby the protein content of chicken breast decreases during spoilage (*27*). Table 1 shows that the stored control had the lowest protein mass fraction at the end of the 14-day storage period, which was a decrease of 5.63 % from the initial value of the fresh sample.

The treated samples all had higher protein mass fraction on day 14 than the stored control, but only the protein mass fractions of the samples treated with 1 and 1.5 mL West Indian bay leaf essential oil and samples treated with 1 mL essential oil mixture were significantly different (p<0.05) from the stored control. Furthermore, the sample treated with 1 mL West Indian bay leaf essential oil had the highest protein mass fraction of all the treated samples and was not statistically different (p>0.05) from that of the fresh sample, indicating that it could reduce the protein loss of the sample by 98.9 % compared to the refrigerated control.

The ability of an essential oil to prevent protein oxidation and degradation depends largely on its phenolic content (*28*). In this study, the higher TPC of West Indian bay leaf essential oil enabled better preservation of protein content in the samples. Al-Hijazeen (*28*) showed that the rate of protein oxidation decreased as more essential oil was used on a sample, and thus became exposed to more phenolic substances, which effectively attenuated the changes in protein content. This trend was not consistent for the bay leaf samples, as the retained protein initially increased with volume increase from the 0.5 to 1 mL, but then decreased at the volume of 1.5 mL. Conversely, the trend was consistent for the turmeric samples, as the values increased with higher volumes. However, none of the values obtained were significantly different (p>0.05) from each other.

The effects of the mixed essential oil treatments were not enhanced, as the protein mass fraction of the samples was not significantly different (p>0.05) from those of the single oils at any of the corresponding volumes. Thus, in terms of protein oxidation, West Indian bay leaf and turmeric essential oil did not have any synergistic or additive effect when used together.

### Effect of essential oil on moisture content

The moisture content of the sample of the original fresh chicken breast was close to the values (72−74 %) determined in a previous study (*27*). It was expected that the moisture content of the samples would decrease rapidly by day 14 due to evaporation during prolonged storage and the loss of water-holding capacity (WHC) that occurs during microbial and autolytic protein degradation, which causes pH changes (*29*). As shown in Table 1, all samples had a final moisture mass fraction in the range of 23.3–28.5 %, a decrease of about one third of the moisture mass fraction of the original fresh chicken breast sample. All treated samples had a higher moisture mass fraction at the end of refrigerated storage than the refrigerated control. The sample treated with 1.5 mL of mixed essential oils had the highest moisture mass fraction compared to the other treatments.

Heydari *et al.* (*30*) showed a linear relationship between pH and WHC, *i.e.* as the pH decreased, the WHC of the meat also decreased, resulting in higher water loss and lower moisture content of the sample. This resulted in protein structure loss that occurred with a change in pH and reduced the efficacy of the water binding capacity. However, in this study, this expected trend between the pH and moisture mass fraction of the samples was generally not observed, except for the treatment with turmeric oil. As mentioned above, only the pH of the turmeric samples increased with the increase of oil volume (from 0.5 to 1.5 mL). This trend of pH increase with simultaneous increase in moisture mass fraction of the turmeric samples was also reported by Hedyari *et al.* (*30*).

The higher protein mass fraction in the treatments with 1 and 1.5 mL of West Indian bay leaf essential oil and with 1 mL of essential oil mixture was expected to contribute to a higher WHC and thus these samples were expected to have higher moisture mass fraction. This was observed, but these higher values were not statistically significant (p>0.05). However, the treatment with 1.5 mL of essential oil mixture resulted in the highest moisture mass fraction (p<0.05). The effect of the oil on moisture retention was stronger when a 1.5 mL of oil mixture (0.75 mL of each bay leaf and turmeric essential oil) was used than with the individual volumes of 1 and even 1.5 mL.

### Effect of essential oil on sample colour

Vital *et al.* (*31*) showed that the L* value (lightness) of beef samples decreased during storage, but it was lower in the samples treated with rosemary and oregano essential oil. They attributed this decrease to structural changes in the meat proteins that are oxidised during storage, which can lead to increased light scattering, and thus a decrease in the overall lightness of the sample. Most likely, the antioxidant and antibacterial effects of the administered oils reduced the protein structural changes, resulting in a smaller decrease in the L* value. Similarly, on day 14, all treated samples had significantly higher (p<0.05) L* values than the refrigerated control (Table 1). For each type of oil treatment, addition of 0.5 mL resulted in the lowest L* values, but as the volume of each oil increased, the L* values increased significantly (p<0.05), with the samples treated with 1.5 mL of West Indian bay leaf oil and 1 mL of essential oil mixture having the highest values. As more oil was used, fewer protein structural changes occurred, resulting in lower light scattering and a higher L* value. Furthermore, the samples treated with 1 and 1.5 mL of essential oil mixture had significantly higher (p<0.05) L* values than the samples treated with the corresponding single oils, indicating that the treatment with oils mixture had a stronger effect on the lightness of the samples.

During meat spoilage, a characteristic greenish colour develops, which is partly due to the microbial production of hydrogen sulphide, hydrogen peroxide and sulphomyoglobin (*1*). In this study, it was expected that the essential oil would prevent or at least minimise this colour change by inhibiting microbial growth, and that the stored control sample would show this expected colour change. The a* values of the samples measured the degree of redness (positive a* values) or greenness (negative a* values). Table 1 shows that although the a* values of all stored samples decreased compared to the fresh control, none of the samples showed negative values (green hue) indicating the presence of sulphomyoglobin. Although the refrigerated control had the lowest a* value, which was twice as low as the initial value of the fresh sample, it still did not show the expected green colour. This indicates that even after 14 days of storage, green-coloured sulphomyoglobin was not formed.

As already mentioned, the redness of the samples decreased, indicating that the initial protein oxymyoglobin, that gives bright red colour to the fresh samples, was transformed during storage into the duller, reddish-brown metmyoglobin pigment. This occurred because the iron from the haeme group of the protein-pigment complex was oxidized by prolonged exposure to atmospheric oxygen, resulting in a colour change (*32*). Apart from the sample treated with 1.5 mL of essential oil mixtures, the samples all had significantly higher a* values (p<0.05) than the refrigerated control. These results support a previous study showing that the antioxidant properties of the essential oil were able to delay the oxidation of the haeme group, allowing the treated meat samples to show more stable colours (*28*). Although the treatment with 1.5 mL of turmeric essential oil showed the highest a* values, no clear trend was observed across the administered volumes of each type of oil treatment. Additionally, no clear trend of a synergistic, enhancing effect was observed when the individual oils were used in combination, as only the treatment with 1 mL of oil mixtures had a higher a* value compared to the treatment with 1.5 mL.

All samples showed increased b* values at the end of storage compared to the fresh sample. However, when the volumes of each oil increased from 0.5 to 1.5 mL, no distinct, uniform trend was observed as the values fluctuated. As a result, the L* and a* values were used to give insight into how the essential oil affected the colour of the samples during storage.

### Microbiological assessment of samples

#### Total plate count

The total plate colonies of the fresh sample (Table 2) were considered too few to count (TFTC), while the stored control was deemed too numerous to count (TNTC) at the end of the storage period. Similarly, all samples treated with turmeric essential oil were considered TNTC at the end of storage, while the majority of the other essential oil samples had lower total bacterial loads than the stored control sample.

**Table 2:** Microbiological load of fresh control (day 0 at 28 °C), refrigerated control (day 14) and treated (day 14) chicken breast samples stored at 4 °C

Sample treatment	*N*(total plate)/(CFU/g)	*N*(lactic acid bacteria)/(CFU/g)	*N*(yeast and mould)/(CFU/g)
*V*/mL Turmeric oil			
0.5	TNTC	2.43·10^5^	6.95·10^6^
1	TNTC	1.09·10^5^	1.27·10^7^
1.5	TNTC	TFTC	TFTC
West Indian bay leaf oil			
0.5	TNTC	1.01·10^7^	1.80·10^7^
1	1.66·10^6^	1.43·10^5^	1.88·10^5^
1.5	1.37·10^6^	8.50·10^4^	1.60·10^5^
Oil mixture			
0.5	1.27·10^8^	4.65·10^6^	6.30·10^6^
1	2.62·10^7^	2.23·10^6^	3.60·10^5^
1.5	1.10·10^7^	TFTC	5.70·10^4^
*t*/day			
0 (fresh control)	TFTC	TFTC	TFTC
14 (refrigerated control)	TNTC	TNTC	TNTC

TNTC=too numerous to count, TFTC=too few to count

The total phenolic content (TPC) of the essential oil has been shown in previous studies to be the main reason for its antibacterial activity (*22*). Since West Indian bay leaf essential oil had a much higher TPC than turmeric essential oil, it is understandable that these samples had much lower CFU/g values than the turmeric essential oil samples. The sample treated with 1.5 of mL West Indian bay leaf essential oil had the lowest count. As the volume of bay leaf essential oil increased, the CFU/g values decreased further due to exposure to the higher TPC, which exerted a stronger antibacterial effect. It was not observed that the effect of the oils was enhanced when used in mixture, as the samples did not show lower bacterial loads than the samples treated with the bay leaf essential oil, although the bacterial load decreased with higher volumes of the oil mixtures. It can therefore be assumed that the addition of the ineffective turmeric essential oil had a diminishing effect on the efficacy of the West Indian bay leaf essential oil when they were used in mixture.

#### Lactic acid bacteria

Table 2 shows that all treated samples had a much lower lactic acid bacteria (LAB) load than the stored control, which had a load considered to be TNTC. This observation is supported by the results of a previous study which showed that the presence of oregano oil (1 %) in combination with modified atmosphere packaging was able to keep the initial LAB load in a chicken breast sample relatively constant at 3.66 CFU/g even after 15 days of storage, while the load in the control sample approximately doubled after only six days (*33*).

While the total plate count results showed that turmeric essential oil was ineffective in reducing the total aerobic bacterial load of the samples, it was found to be most effective against LAB. As shown in Table 2, the antibacterial efficacy of the essential oil was clearly evident as all treated samples had a much lower LAB load than the stored control. At each volume, the samples treated with West Indian bay leaf essential oil had a higher LAB load than the samples treated with turmeric essential oil, suggesting that the former was not as effective in reducing the LAB growth. As observed earlier, turmeric essential oil had a higher total flavonoid content (TFC) than West Indian bay leaf essential oil, including various curcuminoids (*11*). Curcuminoids such as curcumin have been shown to be very effective against Gram-positive bacteria (*34*) so that the proliferation of Gram-positive LAB in the chicken breast samples would have been effectively inhibited after exposure to turmeric essential oil.

This observed trend of turmeric oil proving to be a more effective oil treatment could explain the observed trends in pH results. While the general trend was that as the volume of essential oil increased, the corresponding pH decreased, this was not observed in the samples treated with turmeric oil. These samples showed slightly increasing pH values as more oil was used. This could be partly due to the fact that turmeric essential oil does not inhibit the growth of proteolytic bacteria as effectively as the other oils, but also due to an effective inhibition of LAB growth in the sample. Since LAB growth was lower in the samples treated with turmeric oil, this would mean that the LAB-induced decrease in pH during storage would also be lower and the samples would have slightly higher pH values than the others. The use of oil mixtures was effective, as shown by the low bacterial load, but not more effective than the individual oils.

#### Yeasts and moulds

The ability of the essential oil to delay yeast and mould growth has been observed previously (*35*), where the presence of the essential oil reduced the yeast and mould count of treated samples by at least 50 % at the end of storage compared to control samples. As observed for LAB, since yeast and mould are usually found in spoilt meat samples, their load in the fresh sample was considered to be TFTC, while the load in the refrigerated control was considered to be TNTC.

In general, treatment with turmeric essential oil was the most effective against yeasts and moulds, as it was the only one (at the volume of 1.5 mL) that reduced the number of yeasts and moulds to TFTC. Gul and Bakht (*12*) reported that higher volumes of turmeric essential oil reduced the fungal counts by almost 50 % in treated samples compared to the untreated ones. It was also shown that turmeric essential oil contains saponins in addition to flavonoids and phenols (*36*). The study also showed that saponins are active antifungal compounds, which could explain why the turmeric oil in this study was so effective against yeast and mould in the treated samples. However, in contrast to previous observations, the sample treated with 0.5 mL of turmeric oil had a lower load than the sample treated with a higher volume (1 mL), which was inconsistent and may be due to human error. No major synergistic effect was observed when the mixtures of oils were used.

### Sensory characteristics of the samples

#### Odour ratings

Table 3 shows the odour ratings of the refrigerated control and treated samples on the last day (day 14), with the control receiving an average odour score of 1.0 (unacceptable). With the exception of the samples treated with 0.5 mL of turmeric and 0.5 mL of West Indian bay leaf essential oil, all other treated samples received significantly higher (p<0.05) scores than the control on the last day. Due to the antimicrobial properties of the oils highlighted previously, the results showed that the treatments with essential oil (except the treatments with 0.5 mL West Indian bay leaf and 0.5 mL turmeric oil) could have delayed microbial-induced off-odours, as reported by Chouliara *et al.* (*33*). The volume of 1.5 mL of each essential oil generally gave the best results, except in the case of West Indian bay leaf essential oil, where the samples treated with 1 mL of the essential oil received the highest scores with the average score of 3.4 on the last day. The pungency and strong odour of the West Indian bay leaf essential oil at the highest volume (1.5 mL) could explain the lower rating by the panellists. The samples treated with turmeric essential oil received the lowest scores among all treated samples, and although an increase in the volume of oil resulted in a slight increase in the scores at the final day, the ratings were not significantly different among the volumes (p>0.05).

**Table 3:** Sensory scores of chicken breast samples stored at 4 °C on the last day of storage

Treatment	*V*(oil)/mL	Odour	Colour	Appearance	Overall acceptability
Turmeric oil	0.5	(1.5±0.6)^ab^	(1.5±0.7)^a^	(1.4±0.5)^a^	(1.5±0.6)^ab^
	1	(1.7±0.8)^b^	(1.8±0.9)^a^	(2.1±0.9)^b^	(1.9±0.7)^bcd^
	1.5	(1.9±0.9)^b^	(1.5±0.6)^a^	(1.6±0.7)^a^	(1.6±0.7)^abc^
West Indian bay leaf oil	0.5	(1.5±0.8)^ab^	(2.5±0.9)^b^	(2.1±0.8)^b^	(2.1±0.8)^cd^
	1	(3.4±0.9)^de^	(2.7±0.8)^b^	(2.6±0.6)^c^	(3.0±0.8)^e^
	1.5	(2.9±0.9)^cd^	(1.9±0.8)^a^	(1.8±0.8)^ab^	(2.4±0.7)^d^
Oil mixture	0.5	(1.8±0.8)^b^	(1.8±0.8)^a^	(1.5±0.6)^a^	(1.9±0.8)^bcd^
	1	(2.6±0.8)^c^	(1.7±0.8)^a^	(1.8±0.7)^ab^	(1.9±0.6)^bcd^
	1.5	(3.6±1.0)^e^	(2.7±0.6)^b^	(3.1±0.8)^d^	(3.4±0.9)^e^
Refrigerated control (*t*=14 day)	0	(1.0±0.0)^a^	(1.4±0.5)^a^	(1.4±0.5)^a^	(1.3±0.5)^a^

Different letters in superscript in the same column indicate significantly different values (p<0.05)

Heydari *et al.* (*30*) reported in a similar study that samples treated with higher concentrations of essential oil were generally rated better by the panellists. This trend was only observed for the treatments with the essential oil mixtures, as the final score increased with increasing volumes. The samples treated with 0.5 mL of oil mixture received a low score of 1.8, possibly due to the small amounts of each oil used (0.25 mL each of West Indian bay leaf and turmeric essential oil), but it was observed to still slightly outperform the individual 0.5 mL of turmeric and West Indian bay leaf essential oils, both of which received scores of 1.5 at the end of storage. The samples treated with 1.5 mL of oil mixture not only received the highest score among all treated samples (3.6), but also a significantly higher score (p<0.05) than the samples treated with 1.5 mL of individual oils, suggesting that consumers found the odour of the samples more appealing when the oils were used in mixture.

### Colour parameters

Similar to the odour results, the refrigerated control samples received the lowest ratings by the panellists for the colour appearance, with an average score of 1.4 on the last day. Only the samples treated with 0.5 and 1 mL of West Indian bay leaf essential oil and 1.5 mL of the essential oil mixture received significantly higher (p<0.05) colour scores than the control on the last day. Heydari *et al.* (*30*) found that although the control sample in their study had the lowest colour score, the colour scores for the treated samples increased as more essential oil was used. This trend was not observed in this study, as no significant correlation was found between the oil volumes and the colour ratings of samples on the final day. The reason for this could be that in the previous work a colourless essential oil (lavender oil) was used, while in this study we have used oils that were predominantly yellow in colour. The yellow colour of the oils would have given the samples a similar yellow hue and could have influenced the panellists’ ratings. This would explain why the samples treated with the highest volume (1.5 mL) of West Indian bay leaf and turmeric essential oil received the lowest scores, as the amount of yellow colour imparted to the samples was considered undesirable and associated with spoilage by the panellists. Furthermore, as the mixture of 1.5 mL of essential oils contained only 0.75 mL of each oil, it was rated highly, as the samples were less yellow.

### Appearance of meat samples

As the meat samples spoiled during storage, their appearance was undesirable, mainly because of surface slime and mould growth that occurred in some cases. The refrigerated control sample received the lowest rating from the panelists on the last day. Only the samples treated with 1 mL of turmeric essential oil, 0.5 and 1 mL of West Indian bay leaf essential oil, and 1.5 mL of essential oil mixture received significantly higher (p<0.05) ratings.

For the individual West Indian bay leaf and turmeric essential oil treatments, the samples treated with 1 mL received a significantly higher (p<0.05) appearance scores on the last day than the samples treated with other oil volumes. The volume of 1.5 mL did not receive significantly higher (p>0.05) scores than the 0.5 mL, which probably meant that 0.5 mL was too low to prevent microbial-induced changes in appearance, while 1.5 mL was too high and would have given panellists some unfavourable changes, such as increased oily texture of the sample.

However, similar to the previous sensory results, the treatment with 1.5 mL of essential oil mixture received the highest scores on the last day, which were significantly higher (p<0.05) than the highest rated individual oil treatments on the final day. This showed that the use of smaller amounts of the oil mixtures (0.75 mL each) was effective in reducing the microbial-induced changes in appearance without imparting an undesirable oily texture to the samples.

### Overall acceptability

The overall acceptability of the samples was perhaps one of the most important sensory parameters analysed, as it considered the previous sensory parameters and gave an insight into the overall perception of quality, including shelf life, based on the panellists’ scores on the last day of storage. As expected, the refrigerated control received the lowest score of 1.3 on the last day, indicating that it was perceived as unfit for consumption by the panellists. As can be seen in Table 4, the refrigerated control samples received a spoilage rating of less than 3.0 on day 8 of storage, while 50 % of the treated samples received a higher score.

**Table 4:** Overall acceptability scores of refrigerated control (day 14) and treated (day 14) chicken breast samples stored at 4 °C

				*V*(essential oil)/mL						
Time/day	0.5	1	1.5	0.5	1	1.5	0.5	1	1.5	Control
	Turmeric oil			West Indian bay leaf oil			Oil mixture			
0	(7.0±0.0)^a^	(7.0±0.0)^a^	(7.0±0.0)^a^	(7.0±0.0)^a^	(7.0±0.0)^a^	(7.0±0.0)^a^	(7.0±0.0)^a^	(7.0±0.0)^a^	(7.0±0.0)^a^	(7.0±0.0)^a^
2	(6.1±0.5)^de^	(6.4±0.6)^e^	(6.2±0.6)^de^	(5.7±0.5)^bc^	(5.5±0.6)^b^	(5.4±0.5)^b^	(5.9±0.6)^cd^	(5.7±0.6)^bc^	(5.9±0.6)^cd^	(5.0±0.5)^a^
4	(4.7±0.7)^bcd^	(5.1±0.6)^def^	(5.5±0.5)^f^	(4.5±0.5)^bc^	(3.9±0.8)^ag^	(3.6±0.6)^g^	(5.4±0.6)^ef^	(4.9±0.6)^cd^	(5.0±0.6)^de^	(4.3±0.6)^ab^
6	(3.0±0.6)^a^	(3.8±0.6)^cd^	(4.0±0.6)^d^	(4.2±0.6)^d^	(3.5±0.8)^bc^	(2.9±0.8)^a^	(4.0±0.8)^d^	(4.1±0.7)^d^	(4.9±0.6)^e^	(3.1±0.4)^ab^
8	(2.5±0.5)^ab^	(2.7±0.7)^abc^	(2.7±0.6)^abc^	(3.7±0.6)^e^	(3.0±0.6)^bcd^	(2.4±0.5)^a^	(3.2±0.7)^cde^	(3.4±1.0)^de^	(3.5±1.1)^de^	(2.3±0.8)^a^
10	(2.0±0.6)^bc^	(2.4±0.7)^cd^	(2.0±0.6)^a^	(2.4±0.7)^cd^	(2.8±0.5)^d^	(2.0±0.6)^bc^	(2.8±0.4)^d^	(2.4±0.6)^cd^	(3.4±0.7)^e^	(1.7±0.5)^ab^
12	(1.4±0.5)^a^	(2.3±0.8)^b^	(1.6±0.6)^a^	(2.2±0.6)^b^	(2.8±0.5)^c^	(2.3±0.7)^b^	(2.2±0.5)^b^	(2.3±0.6)^b^	(3.5±0.9)^d^	(1.4±0.6)^a^
14	(1.5±0.6)^ab^	(1.9±0.7)^bcd^	(1.6±0.7)^abc^	(2.1±0.8)^cd^	(3.0±0.8)^e^	(2.4±0.7)^d^	(1.9±0.8)^bcd^	(1.9±0.6)^bcd^	(3.4±0.9)^e^	(1.3±0.5)^a^

1=extremely unacceptable, 2=moderately unacceptable, 3=slightly unacceptable, 4=neutral, 5=slightly acceptable, 6=moderately acceptable, 7=extremely acceptable. Different letters in superscript in the same row indicate significantly different values (p<0.05)

With the exception of the samples treated with 0.5 and 1.5 mL of turmeric essential oil, all treated samples received a significantly higher (p<0.05) scores than the control on the last day. This observation is supported by previous work regarding overall acceptability, where meat samples treated with essential oil were consistently rated significantly better than untreated control samples during extended storage (*37*). The highest rated samples were those treated with 1 mL of West Indian bay leaf and 1.5 mL of essential oil mixture, both of which were rated significantly higher (p<0.05) than the other treatments and were not perceived as spoilt by the panellists at the end of the storage period. As the control was considered spoilt by the panellists on day 8, these treatments with essential oil extended the shelf life of the samples by a further 6 days.

## CONCLUSIONS

The results of the study indicate that the essential oils of West Indian bay leaf and turmeric can be used as potential natural meat preservatives. The oils were found to have significant effects on the quality parameters and shelf life of the stored chicken breast samples. Regarding the physicochemical properties of the samples treated with essential oil, although the oils were ineffective on moisture content, there was a significant effect (p<0.05) on the other properties. All treatments significantly delayed pH changes, while all treatments except the treatments with 0.5 and 1.5 mL of essential oil mixture significantly delayed texture and colour changes, respectively. The treatments with 1 and 1.5 mL of West Indian bay leaf essential oil, and 1 mL of essential oil mixture were the only ones that significantly reduced the loss of proteins.

The microbiological analyses showed that the oils generally reduced the microbial load of the samples at the end of storage, with turmeric essential oil proving more effective than West Indian bay leaf essential oil against lactic acid bacteria, yeast and moulds, but ineffective in the reduction of total plate count. In addition, sensory analysis showed that the average hedonic ratings of the treated samples on the final day (day 14) were predominantly higher than those of the control, with the treatments with 1.5 mL of essential oil mixture and 1 mL of West Indian bay leaf essential oil proving to be the most effective. These volumes were rated highest for ‘overall acceptability’ and were found to extend the shelf life and acceptability of the samples by six more days compared to the stored control. The results of this study clearly demonstrate the meat preserving potential of West Indian bay leaf and turmeric essential oils when used individually or as a mixture.
